# Serum RGC-32 in children with systemic lupus erythematosus

**DOI:** 10.1038/s41598-023-38092-y

**Published:** 2023-07-08

**Authors:** Bingxue Huang, Dan Feng, Xiaoling Niu, Wenyan Huang, Sheng Hao

**Affiliations:** Department of Nephrology, Rheumatology and Immunology, Shanghai Children’s Hospital, School of Medicine, Shanghai Jiao Tong University, Shanghai, 200062 China

**Keywords:** Systemic lupus erythematosus, Pathogenesis

## Abstract

Childhood-onset systemic lupus erythematosus (SLE) can be more severe than adult patients. Early diagnosis and accurate evaluation of the disease are very important for the patients. Response gene to complement-32 (RGC-32) protein is the downstream regulator of C5b-9 complex which is the terminal pathway of complement activation. Complement system plays a very important role in the pathogenesis of SLE. RGC-32 in patients with SLE has not been reported yet. We aimed to examine the clinical value of RGC-32 in children with SLE. A total of 40 children with SLE and another 40 healthy children were enrolled for this study. Clinical data were obtained prospectively. Serum RGC-32 was determined by ELISA. We found that serum RGC-32 was significantly elevated in children with SLE than that in the healthy group. Serum RGC-32 was significantly higher in the children with moderately/severely active SLE than that in the children with no/mildly active SLE. Furthermore, serum RGC-32 level correlated positively with C-reactive protein, erythrocyte sedimentation rate and ferritin and correlated negatively with white blood cell counts and C3. RGC-32 may be involved in the pathogenesis of SLE. RGC-32 might become a good biomarker in the diagnosis and evaluation of SLE.

## Introduction

Systemic lupus erythematosus (SLE) is a multisystem autoimmune disease in children affecting skin, serosa, joints, kidney, central nervous system and nearly every organ. Its etiology is complicated^1^. The prevalence of SLE varies according to genetic background and living environment. The global average prevalence rate is 12/100,000–39/100,000. Childhood-onset SLE accounts for about 20% of all SLE patients, with most cases diagnosed between the ages of 12 and 14 years. However, diagnosis before the age of 5 years is rare. Childhood-onset SLE patients may experience more severe symptoms and the mortality rate is higher than that of adults^2,3^. Early diagnosis and correct assessment of the disease severity are crucial for effective management of SLE. Many studies have been conducted in recent years to investigate the pathogenesis of SLE and identify biomarkers that can specifically evaluate disease activity.

Response gene to complement-32 (RGC-32) is an important complement response gene. It has been found that RGC-32 protein may play an extremely important role in cell cycle regulation, complement-mediated inflammatory response, tumor metastasis, tissue cell differentiation and tubulointerstitial fibrosis^4^. Numerous studies on the pathogenesis of SLE indicate that abnormalities in complement activation and immune complex (IC) clearance serve as the basis^5^. RGC-32 is the downstream regulator of C5b-9 complex which is the terminal pathway of complement activation. Furthermore, RGC-32 is relevant to the differentiation of Th17 cells which is involved in the pathology of SLE. In vitro, RGC-32 promotes the differentiation of human Th17 cells, suggesting that RGC-32 signaling may enhance disease expression in SLE by promoting abnormalities in the Th17 pathway^4^. Thus, RGC-32 may be implicated in the pathogenesis of SLE. The purpose of this study is to explore the clinical value of RGC-32 protein in evaluating the disease activity of SLE.

## Methods

### Study designs and participants

Patients admitted to Shanghai Children’s Hospital from September 2019 to December 2020 were enrolled. All the patients met the Systemic Lupus International Collaborating Centers (SLICC) criteria for SLE in 2012. Another 40 healthy children matched with the age and gender of the SLE group who went through routine medical examination in the same period were selected as the healthy control group. The laboratory and clinical data including gender, age, clinical symptoms, systemic lupus erythematosus disease activity index-2000 (SLEDAI-2K) score, complete blood count, erythrocyte sedimentation rate (ESR), ferritin, complement, autoantibodies were collected. The diagnosis of lupus nephritis was based on the American College of Rheumatology (ACR) guideline in 2012 (more than 0.5 g of proteinuria per day or greater than 3 + by dipstick, and/or the presence of cellular casts, including red blood cells, hemoglobin, granular, tubular, or mixed in urinary sediment)^6^.

### Experimental detection

Peripheral venous blood samples were collected from the patients and healthy control group. The serum RGC-32 level was detected by Human Response gene to complement 32 protein(C13orf15) ELISA kit (Cusabio biotech). The operation process was strictly in accordance with the instructions of the kit.

### Statistical analysis

SPSS 23.0 software was used for data processing and statistical analysis. The counting data was presented as frequency and rate, and the *χ*2 test was used for comparative analysis. The continuous data conforming to the normal distribution were represented as mean and standard deviation, and the independent sample *t* test was used for comparison. The continuous data that did not conform to the normal distribution were represented as median and quartile M (Q1, Q3), and the Mann–Whitney test was used for comparison. Spearman correlation analysis was used to analyze the correlation between indicators. The diagnostic value of RGC-32 in SLE was analyzed by receiver operating characteristic curve (ROC curve). *P* < 0.05 was considered statistically significant.

### Ethical approval and informed consent

This study was reviewed and approved by the Ethics Review Committee of Shanghai Children’s Hospital (NO. 2020R015-E01). All methods were performed in accordance with the ethical standards of our institutional research committee and with the 1964 Helsinki declaration. Written informed consent was obtained from the participants’ legal guardian.

## Results

### Baseline information

In this study, a cohort of 40 children diagnosed with SLE was analyzed. Of these patients, 9 were male and 31 were female, with females accounting for 77.5% of the total patient population. The clinical data of the patients was presented in Table 1. The average age of the patients was 11.88 ± 3.11 years old. Among the patients, 7 had fever, 11 presented rash, 5 presented alopecia, 2 presented oral ulcer, 2 presented serositis, 1 presented neurologic symptoms, and 2 presented synovitis. 21 patients had proteinuria, while 18 had hematuria.

**Table 1:** General clinical data of the Children of SLE.

Indicator	Value
Female/n (%)	31 (77.5)
Age/year ($\overline{X }$±s)	11.88 ± 3.11
Fever/n (%)	7 (17.5)
Rash/n (%)	11 (27.5)
Alopecia/n (%)	5 (12.5)
Oral ulcer/n (%)	2 (5)
Serositis/n (%)	2 (5)
Neurologic symptoms/n (%)	1 (2.5)
Synovitis/n (%)	2 (5)
Proteinuria/n (%)	21 (52.5)
Hematuria/n (%)	18 (45)

*SLE* systemic lupus erythematosus.

### Level of RGC-32 in children with SLE and healthy control group

The expression of RGC-32 protein in 40 patients and 40 healthy children was detected. The serum concentrations of RGC-32 between the two groups were shown in Table 2. A significant difference was detectable. The level of RGC-32 in children with SLE was 326.12 (212.47, 753.43) pg/ml, and that of healthy controls was 148.29 (110.28, 178.46) pg/ml. RGC-32 expression was higher in patients with SLE (*Z* = − 4.657, *P* < 0.001).

**Table 2:** Comparison of RGC-32 between SLE group and control group.

Item	SLE group (n = 40)	Control group (n = 40)	*Z* value	*P* value
RGC-32/pg.mL^-1^	326.12 (212.47, 753.43)	148.29 (110.28, 178.46)	− 4.657	< 0.001*

*RGC-32* response gene to complement-32, *SLE* systemic lupus erythematosus. Data are represented as interquartile range and the Mann–Whitney test is used for comparison.

*P value below 0.05 indicates statistical significance.

### Analysis of diagnostic value of RGC-32 in SLE

**Figure 1:**
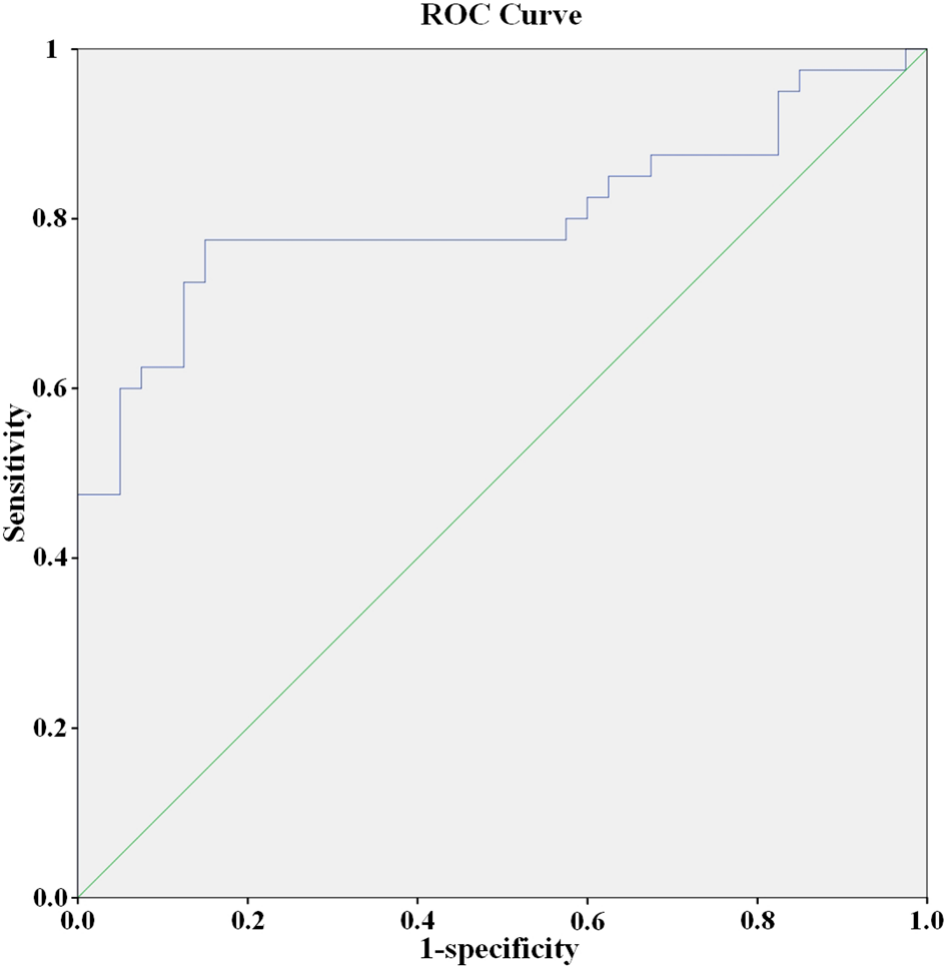
ROC curve of RGC-32 in diagnosing patients with SLE.

The area under the curve (AUC) of RGC-32 for the diagnosis of SLE was 0.803 (95% CI 0.699–0.906) (*P* < 0.05, Fig. 1). Based on the sensitivity and (1- specificity) of the coordinates of the ROC curve, the Youden index was calculated. The cutoff value of RGC-32 in the diagnosis of SLE was 206.4 pg/mL. When the expression level of RGC-32 was more than 206.4 pg/mL, the sensitivity and specificity for the diagnosis of SLE were 77.5% and 85%.

### Level of RGC-32 in patients between different subgroups

Among the 40 patients, a total of 39 patients’ laboratory data were complete. There were 21 cases with no/mild activity (SLEDAI < 10) and 18 cases with moderate/severe activity (SLEDAI ≥ 10). Comparison of serum RGC-32 in these two groups was made. The serum level of RGC-32 was 275.84 (114.86, 459.47) pg/ml in no/mildly active group and 528.07 (235.47, 1010.00) pg/ml in moderately/severely active group. RGC-32 was significantly higher in the moderately/severely active group than that in no/mildly active group (*Z* = 2.217, *P* = 0.027).

There were 16 cases with positive anti-dsDNA antibody and 23 cases with negative result. Comparison of serum RGC-32 in these two groups was made. The serum level of RGC-32 in patients with positive anti-dsDNA antibody was 522.18 (332.15, 948.78) pg/ml and that in patients with negative anti-dsDNA antibody was 226.12 (125.31, 623.00) pg/ml. RGC-32 was significantly higher in the positive group (*Z* = 2.712, *P* = 0.007).

Meanwhile, there were 23 cases with lupus nephritis and 16 cases without lupus nephritis. However, the level of RGC-32 in patients without lupus nephritis was 326.12 (242.00, 592.00) pg/ml and that in children with lupus nephritis was 322.68 (133.16, 778.19) pg/ml. There was no significant difference between the two groups (*Z* = 0.542, *P* = 0.587) (Table 3).

**Table 3:** Comparison of RGC-32 in patients with SLE between different groups.

Clinical feature	n	RGC-32/pg.mL^-1^
SLEDAI		
< 10	21	275.84 (114.86, 459.47)
≥ 10	18	528.07 (235.47, 1010.00)
*Z* value		2.217
*P* value		0.027*
Anti dsDNA antibody		
Positive	16	522.18 (332.15, 948.78)
Negative	23	226.12 (125.31, 623.00)
*Z* value		2.712
*P* value		0.007*
Lupus nephritis		
No	16	326.12 (242.00, 592.00)
Yes	23	322.68 (133.16, 778.19)
*Z* value		0.542
*P* value		0.587

*RGC-32* response gene to complement-32, *SLE* systemic lupus erythematosus, *SLEDAI* systemic lupus erythematosus disease activity Index. Data are represented as interquartile range and the Mann–Whitney test is used for comparison.

*P value below 0.05 indicates statistical significance.

### Correlation analysis between serum RGC-32 level and clinical disease activity index

The correlation between serum RGC-32 and other clinical indexes was analyzed by Spearman correlation analysis. The result was illustrated in Table 4. Serum RGC-32 level correlated positively with CRP, ESR and ferritin (*r* = 0.336, 0.349 and 0.402, *p* = 0.036, 0.030 and 0.012) and correlated negatively with WBC counts and C3 (*r* = − 0.517 and − 0.466, *p* = 0.001 and 0.003). It was not correlated with hemoglobin or platelet (*r* = − 0.291 and 0.004, *p* = 0.072 and 0.979).

**Table 4:** Correlation analysis between RGC-32 and other indicators of activity.

	WBC counts	Hemoglobin	Platelet	CRP	ESR	Ferritin	C3
*r*	− 0.517	− 0.291	0.004	0.336	0.349	0.402	− 0.466
*p*	0.001*	0.072	0.979	0.036*	0.030*	0.012*	0.003*

*RGC-32* response gene to complement-32, *WBC* white blood cell, *CRP* C-reactive protein, *ESR* erythrocyte sedimentation rate. Spearman correlation analysis was used.

*P value below 0.05 indicates statistical significance.

## Discussion

Childhood-onset SLE is known to exhibit greater disease severity than adult-onset SLE. Timely diagnosis and precise disease evaluation are imperative for effective management of the condition. There is a need for reliable, widely applicable and minimally invasive biomarkers to assist in diagnosing the disease and optimizing management. Currently, researches on childhood-onset SLE biomarkers have been active, with multiple gene-based and blood-based biomarkers being investigated, including the interferon signature, methylation status, neutrophil extracellular traps, S100 proteins, and complement split products^7^. These promising biomarkers hold potential for improving the diagnosis and treatment of childhood-onset SLE. However, before they can be implemented in clinical care, further intensive study is required in additional patient cohorts. Therefore, it is necessary to discover new biomarkers that can be combined with existing ones to enhance the accuracy of diagnosis and efficacy of treatment.

Complement system activation is an important feature of SLE. High levels of IC formed by autoantibodies and antigen are widely deposited on the blood vessel walls of glomerulus, joints and other organs in patients with SLE. It strongly activates the complement system and causes inflammatory damage. Therefore, patients with active SLE often show the decrease of serum complement level. The decrease of serum C3 and/or C4 is also one of the disease activity indexes of SLE, which is an item in the SLEDAI score. The different complement activation pathways converge at the activation of C5 and then form the membrane attack complex C5b-9. C5b-9 is involved in the pathogenesis of SLE and lupus nephritis, but the specific mechanism is still unknown.

RGC-32 was first cloned by Beada et al.^8^. It is an important complement response gene. Human RGC-32 gene is located at 13q14.11 and encodes RGC-32 protein, which is distributed in many tissues and organs of human body and participates in normal physiological functions. RGC-32 protein participates in inflammatory progress^9^. Fosbrink et al. showed that C5b-9 can induce the expression of RGC-32^10^. If the RGC-32 gene of human endothelial cells and smooth muscle cells was knocked out in vitro, the cell proliferation mediated by C5b-9 was stopped, suggesting that RGC-32 protein was the downstream regulator of cell proliferation and inflammatory process induced by C5b-9. The results of our study showed that the expression of RGC-32 in pediatric patients with SLE was significantly higher than that in healthy children, especially in children with high disease activity. Meanwhile, anti-dsDNA antibody is often used independently to judge the disease activity of SLE. In this study, it was found that the serum RGC-32 level in the SLE anti-dsDNA positive group was significantly higher than that in the negative group. Therefore RGC-32 is probably involved in the pathogenesis of SLE.

Many studies have found RGC-32 to be implicated in the pathogenesis of a number of other autoimmune diseases. Kim et al. found that the levels of RGC-32mRNA in the skin of psoriatic lesions were significantly lower than those in the skin of healthy individuals^11^. Chen et al. found that RGC-32 may play an important role in regulating the cell cycle progression of chondrocytes in the context of the hypoxic joint microenvironment in patients with rheumatoid arthritis^12^. Thus, RGC-32 may be involved in other autoimmune diseases. The difference of RGC-32 between SLE and other autoimmune diseases needs to be further investigated.

The research on the pathogenesis of SLE has made lots of progress in recent years. But no specific biomarker for SLE has been widely recognized and accepted. C3 is a traditional index of disease activity. However, the level of complement is not only related to the consumption of complement activation, but also to the rate of complement synthesis. In the process of inflammatory reaction, the consumption and synthesis of complement increase significantly in the same time. The product during complement activation is considered as a more specific biological marker of SLE^13^. Many studies have shown that C1r, C1s, C4a-d, Bb, C3a-d, C5a, C5b-9 and other products are more specific to complement activation than C3 and C4^14^. Porcel et al. showed that in 61 SLE patients C5b-9 had the highest correlation with disease activity score among various complement activation products^15^. The research results of Chiu et al. on 41 SLE patients also showed that the levels of C5b-9, C3a and C4 were significantly different between mild and moderate severe SLE patients. Among them C5b-9 was most correlated with the severity of the disease (*P* < 0.0005)^16^. RGC-32 is the downstream regulator of C5b-9 complex, and its expression in SLE has not been reported yet. The results of our study showed that RGC-32 level was correlated positively with C-reactive protein, ESR and ferritin and correlated negatively with WBC counts and C3. We can see RGC-32 is related to some commonly used laboratory indexes that can reflect the disease activity. But the sample size of this study is relatively small and the data do not conform to the normal distribution. Therefore, the results need more data for further verification.

Huang et al. showed that RGC-32 played a critical role in transforming growth factor-β induced epithelial-mesenchymal transition of renal tubular cells^17,18^. Niu et al. found that RGC-32 protein may be involved in the tubulointerstitial lesions of children with IgA nephropathy, especially in the course of the epithelial-mesenchymal transition induced by TGF-β^19^. RGC-32 plays an important role in the occurrence of renal injury. Contrary to expectation, in this study we compared the two groups of patients with and without lupus nephritis, and found that there was no significant difference in serum RGC-32 levels between the two groups. In the future, further research on the expression of RGC-32 protein in kidney is needed to clarify the significance of RGC-32 in the pathogenesis of lupus nephritis.

## Data Availability

The data sets generated during and/or analyzed during the current study are available from the corresponding author on reasonable request.
